# Modified STING-activating cyclic dinucleotide derivatives significantly enhance the anti-tumor activity of therapeutic vaccines

**DOI:** 10.1186/2051-1426-1-S1-O20

**Published:** 2013-11-07

**Authors:** Meredith Leong, David Kanne, Laura Glickman, Edward Lemmens, Peter Lauer, Ken Metchette, Dara Burdette, Elie Diner, Juan Fu, Kevin Soares, Dirk Brockstedt, Daniel Portnoy, Russell Vance, Young Kim, Elizabeth Jaffee, Drew Pardoll, Thomas Dubensky

**Affiliations:** 1 Aduro BioTech, Berkeley, CA, USA; 2 University of California, Berkeley, CA, USA; 3 Sidney Kimmel Cancer Center, Johns Hopkins University, Baltimore, MD, USA

## 

The STING signaling pathway has emerged as a central TLR-independent mediator of host innate defense in response to sensing cytosolic nucleic acids, either through direct binding of CDNs secreted by intracellular bacteria, or through binding of a structurally distinct CDN produced by a host cell receptor, cyclic GMP-AMP (cGAMP) synthase (cGAS), in response to binding cytosolic double-stranded DNA. Binding of CDNs to STING initiates a signaling cascade through the TBK-1/IRF-3 axis to induce type I interferon (IFN) and other co-regulated genes. Discovered recently, CDNs synthesized by cGAS and bacteria are structurally distinct. The cGAS product, termed non-canonical CDN, has a phosphate bridge with both 2’-5’ and 3’-5’ linkages that reportedly increases its affinity for STING by 200-fold. Here we show in a panel of human donors that the non-canonical linkage confers significantly increased activity of CDNs to activate PBMCs, as measured by expression of IFN and NF-κB dependent cytokines, as compared to CDNs with canonical 3’-5’ phosphate linkages. By conducting Immunogenicity-Structure Activity Relationship studies in vitro and in various animal models of infection and cancer, we selected synthetic derivatives of native CDNs produced by bacteria or eukaryotic cells with increased potency. CDN derivatives with sulfur atoms at non-bridging oxygens of the internucleotide phosphate bridge were resistant to digestion with phosphodiesterase. Surprisingly, R,R di-thio CDN diastereomers induced higher levels of IFN in vitro, and induced a higher magnitude of peak and memory antigen-specific CD4 and CD8 T cell responses correlated with protective immunity, as compared to either the R,S di-thio CDN diastereomer, or di-oxo CDN. CDNs based on adenosine nucleotides were comparatively independent of host cell permeabilization to activate STING signaling. We applied this finding to enhance the activity of immunotherapy regimens utilizing irradiated GM-CSF expressing tumor cell vaccines. Co-formulation with R,R dithio-c-di-AMP significantly inhibited tumor growth in several models, correlated with increased mobilization and activation of dendritic cells, increased tumor lymphocyte infiltration, and T cell immunity. Immunologic potency of CDNs was essentially lost in mice encoding a non-functional STING allele. Collectively, CDNs have high translation potential for diverse applications in clinical oncology.

